# Sustainable prevention of obesity through integrated strategies: The SPOTLIGHT project’s conceptual framework and design

**DOI:** 10.1186/1471-2458-12-793

**Published:** 2012-09-17

**Authors:** Jeroen Lakerveld, Johannes Brug, Sandra Bot, Pedro J Teixeira, Harry Rutter, Euan Woodward, Oddrun Samdal, Lynn Stockley, Ilse De Bourdeaudhuij, Patricia van Assema, Aileen Robertson, Tim Lobstein, Jean-Michel Oppert, Róza Ádány, Giel Nijpels

**Affiliations:** 1 The EMGO Institute for Health and Care Research and the departments of General Practice and Epidemiology & Biostatistics, VU University Medical Center, van der Boechorststraat 7, 1081, BT, Amsterdam, the Netherlands; 2 Technical University of Lisbon, Faculty of Human Kinetics, FMH, Estrada da Costa, 1495-688, Cruz Quebrada, Portugal; 3 European Centre on Health of Societies in Transition, London School of Hygiene and Tropical Medicine, 15-17 Tavistock Place, London, WC1H 9SH, UK; 4 European Association for the Study of Obesity, 113-119 High Street, Hampton Hill, Middlesex, TW12 1NJ, UK; 5 Department of Health Promotion and Development, University of Bergen, PO Box 7808, NO-5020, Bergen, Norway; 6 BHF Health Promotion Research Group, Department of Public Health, University of Oxford, Old Road Campus, Headington, Oxford, OX3 7LF, UK; 7 Department of Movement and Sport Sciences, Ghent University, Watersportlaan 2, 9000, Ghent, Belgium; 8 Department of Health Promotion, Maastricht University, PO Box 616, 6200, MD, Maastricht, The Netherlands; 9 Metropolitan University College (Metropol), Pustervig 8, DK-1126, Copenhagen, Denmark; 10 International Obesity TaskForce/International Association for the Study of Obesity, Charles Darwin House, 12 Roger Street, London, WC1N 2JU, UK; 11 University of Paris 13 (UP13), UREN/Center for Human Nutrition Research (CRNH) Ile-de-France, SMBH, 75 avenue Marcel Cachin, 93017, Bobigny, France; 12 Department of Preventive Medicine, University of Debrecen, Medical and Health Science Center, Faculty of Public Health, Kassai Street 26, 4028, Debrecen, Hungary

**Keywords:** Obesity, Prevention, Adults, Environment, Lifestyle behaviour

## Abstract

**Background:**

The prevalence of overweight and obesity in Europe is high. It is a major cause of the overall rates of many of the main chronic (or non communicable) diseases in this region and is characterized by an unequal socio-economic distribution within the population. Obesity is largely determined by modifiable lifestyle behaviours such as low physical activity levels, sedentary behaviour and consumption of energy dense diets. It is increasingly being recognised that effective responses must go beyond interventions that only focus on a specific individual, social or environmental level and instead embrace system-based multi-level intervention approaches that address both the individual and environment. The EU-funded project “sustainable prevention of obesity through integrated strategies” (SPOTLIGHT) aims to increase and combine knowledge on the wide range of determinants of obesity in a systematic way, and to identify multi-level intervention approaches that are strong in terms of Reach, Efficacy, Adoption, Implementation and Maintenance (RE-AIM).

**Methods/Design:**

SPOTLIGHT comprises a series of systematic reviews on: individual-level predictors of success in behaviour change obesity interventions; social and physical environmental determinants of obesity; and on the RE-AIM of multi-level interventions. An interactive web-atlas of currently running multi-level interventions will be developed, and enhancing and impeding factors for implementation will be described. At the neighbourhood level, these elements will inform the development of methods to assess obesogenicity of diverse environments, using remote imaging techniques linked to geographic information systems. The validity of these methods will be evaluated using data from surveys of health and lifestyles of adults residing in the neighbourhoods surveyed. At both the micro- and macro-levels (national and international) the different physical, economical, political and socio-cultural elements will be assessed.

**Discussion:**

SPOTLIGHT offers the potential to develop approaches that combine an understanding of the obesogenicity of environments in Europe, and thus how they can be improved, with an appreciation of the individual factors that explain why people respond differently to such environments. Its findings will inform governmental authorities and professionals, academics, NGOs and private sector stakeholders engaged in the development and implementation of policies to tackle the obesity epidemic in Europe.

## Background

The overall prevalence of overweight and obesity across Europe is high, and has increased dramatically during recent decades in many countries [1]. More than 50% of the total European adult population is now overweight (body mass index (BMI) ≥25) and obesity rates (BMI ≥30) among adults now exceed 20% in many EU Member States [2,3]. Overweight and obesity are, however, not evenly distributed across the European region, with large differences among and within countries, with the problem greatest among those in lower socio-economic groups [3]. Obesity is a major avoidable determinant of the burden of chronic diseases [4,5]. The determinants of obesity - low levels of physical activity, sedentary behaviours and over-consumption of high-energy foods - offer a variety of opportunities for prevention. For decades, approaches to prevention have focused on individual-level determinants, often involving health education approaches [6]. Although more recent developments, such as interventions based on self-determination theory and self-regulatory mediators do offer some promise [7,8], approaches that exclusively target individual-level determinants have had little or no impact on overall rates of obesity and, even those that seem to be effective in small-scale studies have little impact either over the long term or at larger scale [9]. These failures, coupled with innovative methods of geographical research, have led to a recognition that factors in the physical, social-cultural and socio-economic environments at both micro- and macro-levels are driving the obesity epidemic [10-13]. However, while the growth of obesogenic environments may explain trends over time, the distribution of obesity reflects the interaction of environmental and individual factors [14]. The challenge is to combine measures that reduce the obesogenicity of the social and physical environment while, at the same time, reducing the negative impact on individuals of those environments [14,15]. Or, from a health promotion perspective, stimulate individuals' obesity preventing behaviours through environmental facilitation of the behaviours. This will build on recognition that the most effective interventions to prevent obesity a) adopt a system based, integrated, multi-sectoral approach b) involve a complementary range of actions, and c) work at individual and environmental levels through local community interventions and regional/national policy initiatives [16,17]. As has become recognised by agencies such as the World Health Organisation [18], the OECD [19], and researchers across a number of disciplines [20], a system-based, multi-level research approach for obesity prevention frames obesity as a complex systems problem, within which behaviours related to food choices, physical activity and sedentary patterns are not only a matter of individual choice or linear cause and effect, but also strongly influenced by multiple interacting levels of physical, social-cultural and economical environmental factors at micro- and macro-level [15,21]. The different environmental factors can be categorized in the so-called Analysis Grid for Environments Linked to Obesity (ANGELO) [22], which was specifically developed to prioritize environmental factors that relate to obesity (‘obesogenic environments’) in the immediate (micro) and wider (macro) environments, involving physical, economic, policy and socio-cultural dimensions [22,23].

**Figure 1:**
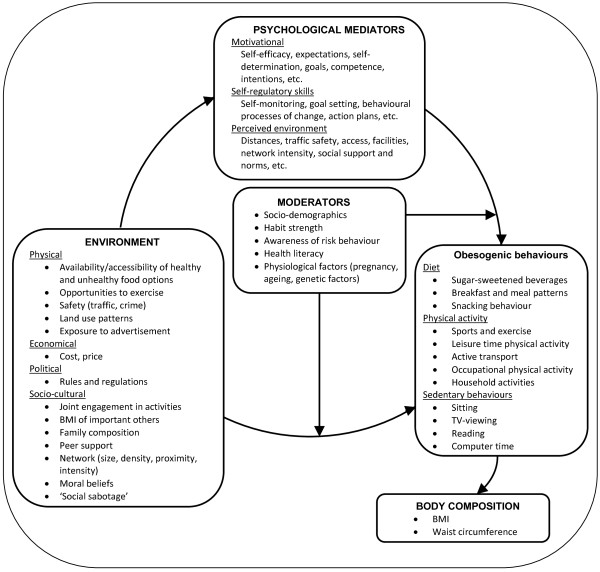
**A SPOTLIGHT specific adaptation of the EnRG (Environmental Research for weight Gain prevention) Framework**[24]

The obesogenicity of an environment can be defined as the sum of influences that the surroundings, opportunities, or wider societal and economic influences have on promoting obesity in individuals or populations [22]. In addition, Kremers et al. [24] argued in their Environmental Research framework for weight Gain prevention (EnRG-) framework that environmental factors (as differentiated in the ANGELO framework) may have a direct impact on obesogenic behaviours, but these environmental influences are also likely to be mediated and moderated by individual-level factors [25]. In the SPOTLIGHT project we have adopted and adapted the EnRG framework (Figure 1), so that it can inform the planned systematic reviews, original cross-European survey research, instrument development and process evaluation research.

There is growing interest in multi-level approaches to obesity prevention [26]. Novel measurement methods can map the obesogenicity of communities [27-29], and projects such as the European Commission-funded HOPE [30] and EURO-PREVOB [31] projects have increased understanding of the broader determinants of obesity. However, there is now a need to consolidate these insights systematically, and to extend, enrich, and operationalise evidence on system-based and integrated approaches at community and societal level. For neighbourhoods, this is of particular relevance to disciplines involved in urban planning, architecture, transportation, and environmental design [32-37]. For national policy-making it develops the evidence base for ‘health in all policies’ and the value of tackling obesity as a multi-sectoral problem involving a wide range of actors in government, commerce, research and professional and public advocacy at national and international level.

The EU-funded SPOTLIGHT project aims to provide a comprehensive overview of the factors on multiple levels necessary to create effective and sustainable interventions to change lifestyles in the real world. These interventions should meet the criteria of Reach (the target population), Efficacy (impact on important outcomes), Adoption (by target settings or institutions), Implementation (consistency of delivery of intervention), and Maintenance (of intervention effects in individuals and settings over time) (RE-AIM) [38,39]. The RE-AIM approach emphasises the external validity of interventions as well as scaling up and sustainability [38,39].

### Objectives of the SPOTLIGHT project

SPOTLIGHT aims to:

I. Identify individual-level, environmental-level and multi-level entry points for approaches aimed at changing obesogenic behaviours and environments

II. Assess interventions using the RE-AIM framework;

III. Identify success-and failure factors for implementation of multi-level intervention approaches;

IV. Provide an evidence-based model for effective multi-level intervention approaches in health promotion practice applicable across the European region, and disseminate the findings to stakeholders in European Union Member States.

## Methods/Design

The methodology to address the aims is summarised below:

I. *Identify individual-level, environmental-level and multi-level entry points for approaches aimed at changing obesogenic behaviours and environments*

The published scientific literature will be systematically reviewed to identify consistent individual-level self-regulation predictors of success in preventing obesity in clinical and community behaviour change interventions. Emphasis will be placed on factors (such as skills, motivation, perceptions, or goals) found to moderate or mediate the effect of interventions on selected outcomes (e.g. body weight, physical activity, sedentary behaviour, eating behaviour). The published literature on both the social and physical environmental determinants of overweight and obesity will also be systematically reviewed. The methodology for conducting the three reviews will follow the guidelines as described in the Cochrane Handbook for Systematic Reviews of Interventions [40]. If appropriate, meta-analytical procedures will be conducted. European as well as non-European studies published since 1995 will be included.

In addition, other methods to address this aim involve 1) the development of an instrument that can be used to assess dimensions of an obesogenic (physical) environment (e.g. walkability, access to food outlets etc.) using remote imaging (data extracted from photographs and geo-localized) and 2) a survey in four countries to assess lifestyle and perceptions of the environment of residents. The work will be conducted in several steps:

Conduct a systematic literature review to identify emerging techniques based on remote satellite imaging to assess measures related to obesogenicity of the environment and the quality of the built environment;

Develop a protocol for data extraction from Google Earth/Streets and test its inter-rater reliability in a selection of neighborhoods in different European countries.

Select 120 neighbourhoods (30 per country, stratified by internationally relevant socio-economic parameters) in four European countries (United Kingdom, Hungary, France and the Netherlands) and conduct a survey on measures related to obesogenicity, using an instrument such as the Environmental Profile of a Community's Health (EPOCH) [41]. Using this instrument has been shown to collect reliable information about the community environment from a variety of settings [41].

Link the environmental obesogenicity measures obtained through remote imaging with collected data on obesity, lifestyle factors and perception of the environment in selected areas in four European Member states.

II. *Assess multi-level intervention approaches using the RE-AIM framework;*

The published scientific literature on the RE-AIM of multi-level intervention approaches to changing obesogenic behaviours in adults will be systematically reviewed. The review will be conducted following the Cochrane methodology. In addition, the RE-AIM of multi-level intervention approaches that are currently being implemented across Europe will be evaluated using a comprehensive cross-European survey. This will result in an interactive web-atlas of multi-level efforts. The following steps will be taken to do this:

Collect existing overviews of recent-and currently implemented multi-level intervention approaches aimed at changing obesogenic behaviours in adults;

Develop a draft atlas of known multi-level intervention approaches, including their general characteristics and their RE-AIM;

Circulate the draft atlas in a small sample of key policy makers and health professionals in a selection of European Member States, and adjust it if needed by adding relevant projects and details;

Further distribute the atlas to policy makers and health professionals in all European Member States (to be reached via the IASO-IOTF and EASO networks) to correct errors and identify any gaps. Accompanying information and instructions will be translated to the relevant national languages of those policy maker and health professionals;

Finalise the web-atlas using the complementary information gathered via the previous step.

III. *Identify success-and failure factors for implementation of multi-level intervention approaches*

A selection of the best examples of effective multi-level approaches in neighbourhoods as identified through the above described methodology will be further studied to address this aim. Quantitative methods will be combined with qualitative methods to reveal factors that enhance or impede implementation and usage of multi-level interventions. The methodology for this has been broken down into the following steps:

Develop a set of parameters to select case studies. The parameters will include intervention characteristics (obesity related, multi-level, integrated), geographical (e.g. Denmark, United Kingdom, Netherlands), and economic status/stage in austerity measures;

Establish which process-frameworks could potentially be used to obtain more in depth insights into determinants that affect the success or failure of implementing multi-level obesity intervention approaches;

Pilot test the developed methodology with an intervention that meets the agreed parameters;

Undertake in-depth research within the selected case study areas with those involved in supporting, delivering, and managing the different levels and components of the intervention to obtain the insights needed e.g. regional and national authorities, community groups, policy makers, nongovernmental organizations, municipal authorities;

If relevant to the interventions selected in the case studies, undertake qualitative research involving consumers;

Compare outcomes between variables – e.g. differences related to geographical characteristics or socioeconomic status.

IV. *Provide an evidence-based model for effective multi-level intervention approaches in health promotion practice applicable across the European region, and disseminate the findings to stakeholders in European Union Member States*

All findings will be translated to a handbook with evidence-based as well as practice-based instructions, suggestions and references to effective and ineffective practice. The dissemination and take-up of findings to the main stakeholders will be facilitated: policy makers, governmental professionals, NGOs, private sector, the scientific community, the media, and other key elements and opinion leaders within the general public. Dialogue with policy-makers will be encouraged through a symposium on the use of evidence in policy-making, and the need for different forms of evidence in the development of health promotion strategies, to be held in the final year of the project. In addition, as the project progresses, a variety of social media platforms (e.g. LinkedIn, Facebook, Twitter) will be considered to encourage discussion of the findings and their relevance among different stakeholders, with opportunities for feed-back to be integrated into SPOTLIGHT outputs. The subject matter should ensure strong interest and engagement across all targeted audiences.

The study protocol was approved by the Medical Ethics Committee of the VU University Medical Center in Amsterdam.

## Discussion

SPOTLIGHT will systematically investigate the determinants of obesity and obesogenic behaviours, multi-level interventions, and factors that enhance adoption of effective interventions in an integrated fashion, applying a range of scientific approaches, including reviews, inventories, tool development, and original data collection. The project aims to improve our understanding of modifiable obesogenic determinants, entry points for intervention approaches and the enhancing and impeding factors for implementation of such measures, applicable across European regions.

The relative recent attention being paid to environmental level determinants in addition to those at individual-level is important for the development of innovative new models for understanding and promoting sustainable prevention of obesity. This is particularly the case with respect to multi-level intervention approaches, which may provide the greatest opportunities for modifying unhealthy lifestyle behaviours across the population, and thus potentially reducing the prevalence of overweight and obesity [25]. In addition, dissemination, implementation or translational research that demonstrates effectiveness in the ‘real world’ is an important next step that is rarely conducted [42,43]. Effective uptake of health promotion interventions will be supported by: understanding how interventions are implemented in the ‘real world’; how to improve the reach of these interventions; how to encourage adoption by individuals, communities and organisations; and finally how to disseminate this understanding [44].

By providing a broad perspective for obesity prevention the project will support the development and implementation of effective obesity prevention approaches by local and national authorities and practitioners across a wide range of disciplines throughout Europe. This perspective will maximise the use of state of the art knowledge and will help policy makers to invest resources in the most effective long-term obesity prevention efforts. In addition, it will provide a direction for health and behavioural scientists to explore further possibilities to reduce obesity, minimize its burden, and decrease the social gradient associated with it and therefore improve the health of European citizens.

## Abbreviations

ANGELO: Analysis Grid for Environments Linked to Obesity; BMI: Body Mass Index; EASO: European Association for the Study of Obesity; EnRG: Environmental Research framework for weight Gain prevention; EPOCH: Environmental Profile of a Community's Health; EURO-PREVOB: Prevention of Obesity in Europe – Consortium for the prevention of obesity through effective nutrition and physical activity actions; GIS: Geographic Information System; HOPE: Health promotion through Obesity Prevention across Europe project; IASO-IOTF: International Association for the Study of Obesitas – International Obesity Task Force; OECD: Organization for Economic Co-operation and Development; RE-AIM: Reach, Effectiveness, Adoption, Implementation, Maintenance; SPOTLIGHT: Sustainable prevention of obesity through integrated strategies.

## Competing interests

The authors declare that they have no competing interests.

## Authors’ contributions

JL, GN, JB and SB designed the study at large and drafted the manuscript. All other co-authors designed (or contributed significantly to) different work packages of the SPOTLIGHT project and provided comments on the draft manuscript. All authors read and approved the final manuscript

## Authors’ information

This paper was published on behalf of the SPOTLIGHT consortium. Information of the full consortium (including biographies) can be found at the projects’ dedicated website: http://www.spotlight-project.eu.

## Pre-publication history

The pre-publication history for this paper can be accessed here:

http://www.biomedcentral.com/1471-2458/12/793/prepub
